# Associations between Adolescents’ Social Leisure Activities and the Onset of Mental Disorders in Young Adulthood

**DOI:** 10.1007/s10964-021-01462-8

**Published:** 2021-06-19

**Authors:** Johanna Timonen, Mika Niemelä, Helinä Hakko, Anni Alakokkare, Sami Räsänen

**Affiliations:** 1 Faculty of Medicine, Research Unit of Clinical Neuroscience, Psychiatry, University of Oulu, Oulu, Finland; 2 Department of Psychiatry, Oulu University Hospital, Oulu, OYS Finland; 3 Faculty of Medicine, Center for Life Course Health Research, University of Oulu, Oulu, Finland

**Keywords:** Adolescents, Mental disorder, Mental health, Social leisure time activity, Community

## Abstract

The association of leisure time activities with specific mental disorders has mainly remained unclear because of lack of such research. This study analyzed the association of different levels of social leisure time activity during adolescence (ages 15–16 years) with the incidence of mental disorders during adolescence and young adulthood between the ages of 16–33 years. The study population consisted of 6838 (females 51.5%) participants from the longitudinal follow-up study, the Northern Finland Birth Cohort 1986, covering 72% of the total 1986 birth cohort. Psychiatric morbidity of the participants was followed through nationwide health registers until the end of the year 2018 when the participants were 33 years old. High social leisure time activity in adolescence associated with lower incidence of psychiatric disorders in general, and specifically, with affective, anxiety, and substance use disorders. Low social leisure time activity was related to increased incidence of any psychiatric disorder in general, and particularly to anxiety and behavioral disorders. In terms of prevention of mental disorders, these study findings encourage families, schools and other communities to continue to enhance and develop community-based social leisure time activities for children and adolescents.

## Introduction

Adolescence is a significant period for psychological development. During this period, leisure time activities make up a significant part of life and they are generally acknowledged to have positive impact on adolescents’ current mental wellbeing (e.g. Rodriguez et al., 2019) However, the relationship between leisure time activity and the development of specific mental disorders has remained unclear. This would be important information for planning and targeting preventive actions during adolescence. The current study examines the longitudinal associations between adolescents’ social leisure activities and the psychiatric diagnoses set in healthcare services by young adulthood.

Leisure time activity is commonly defined as having fun and relaxing activities (Torkildsen, 2005). Involvement in these activities has shown to strengthen adolescents’ physical health, mental well-being, relationship with parents, peer support and perception of academic well-being (e.g., Rochelle et al., 2013). Both cultural and physical activities have been found to be associated with improved self-esteem and psychosocial states, as well as with lower levels of current depressive symptoms and anxiety (Hansen et al., 2015). Accordingly, adolescents with low leisure time activity are reported to have significantly more mental health problems, such as depressive symptoms, anxiety (Bélair et al., 2018), increased substance use and risk behaviors (e.g. Agans et al., 2014), compared to adolescents with active leisure time. The social relationships in leisure time activities have also been emphasized as a possible mediator of the benefits of these activities (e.g., Oberle et al., 2019). Participation in meaningful social leisure time activities has been found to have a positive impact on current and future mental health (Ströhle et al., 2007; Hoegh Poulsen et al., 2016; Doré et al., 2018).

The results of previous studies on the effects of leisure time on mental health remain limited. Most are based on self-assessment questionnaires (see, for example, Badura et al., 2015; Tamminen et al., 2020; Ferreira et al., 2020) or are focus on current mental health issues (e.g. Doré et al., 2016). Research focusing on specific mental disorders conducted in longitudinal study settings is thus mainly lacking.

Limitations in existing research is particularly problematic. That research has demonstrated that different kinds of leisure time activities during adolescence are associated with mental health in general. This knowledge has been an incentive to develop various community-based leisure time models aiming to enhance mental health among youth. Examples of such models are the Icelandic model (Sigfúsdóttir et al., 2011), scout participation (Dibben et al., 2016) and cultural participation (Hansen et al., 2015). However, more research-based information of the relationship of leisure activity and mental health is needed to find effective preventive methods to reverse the alarming rise in the need of psychiatric care for young people.

## Current Study

Research is still limited on whether leisure time activities during adolescence associate with later physician-diagnosed mental health disorders. This research-based information would be of great importance to recognize potential objectives for preventive actions for mental health disorders and thus reduce the need for the use of mental health services. The main objective of this longitudinal study was to examine at epidemiological level the association of socially different leisure time activities at ages 15–16 years with first-onset mental disorders diagnosed in clinical settings at ages 16–33 years. Three specific research questions were addressed. First, the level of social leisure time activity of the study participants was assessed based on a postal survey completed at ages 15–16 years. Second, the psychiatric diagnoses at 16–33 years were extracted from the health care registers. Third, the association of the level of social leisure time activity with first-onset mental disorders was examined after controlling for family-related factors of the study participants.

## Methods

### Study Population

The study population covers the members of the 1986 Northern Finland Birth Cohort Study (NFBC1986). It is an ongoing longitudinal follow-up study including children born in Northern Finland between July 1, 1985 and June 30, 1986. The study involved 99% of the age group of the children born in Northern Finland, of a total of 9432 live-born children. The focus of the NFBC1986 study is to assess morbidity, mortality, and social well-being as well as to identify high-risk groups and possible biological factors that enable risk prevention (Jokela et al., 2019). The NFBC1986 data consisted of postal surveys, clinical trials, and register-based data of the participants as well as parental data based on postal surveys and national registers.

The original cohort study population consisted of 9423 subjects. For follow-up surveys, 9215 cohort members had a known mailing address. Of them, 7344 returned the postal survey administered at the age of 15–16 years. Questions related to hobbies were answered by 7283 cohort members, of whom 7051 gave permission to use their data for research purposes. Of 7051 cohort members, a total of 213 were excluded from this study because of mental disorder diagnoses set before 16 years of age. The most common diagnoses of the excluded participants were behavioral and emotional disorders, with onset usually occurring in childhood and adolescence (F90–F98) (35.2%), mood (affective) disorders (F30–F39) (23.9%), anxiety disorders (F40–F49) (17.4%), and disorders of psychological development (F80–F89) (17.4%).

**Fig. 1:**
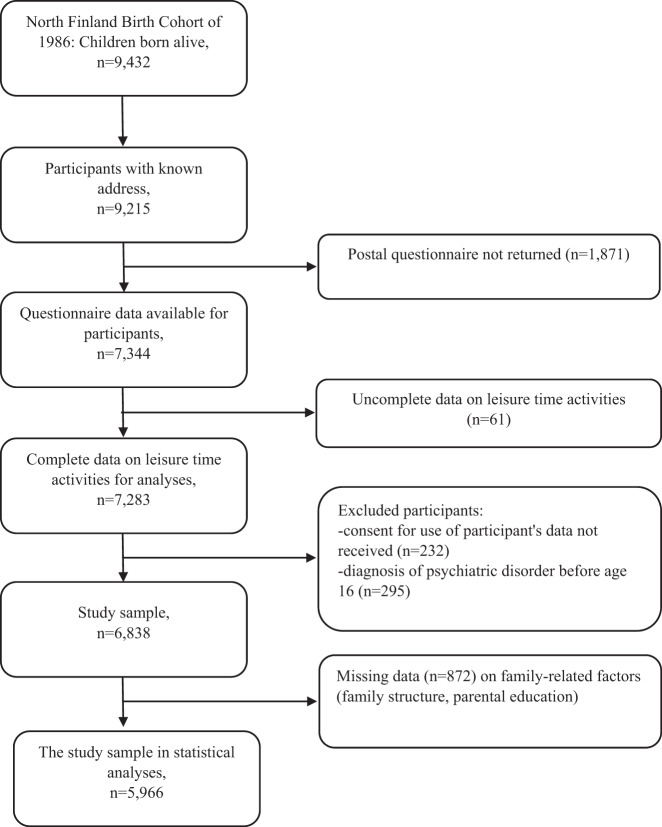
Sampling of the data

The final study population contained 6838 participants, covering 72% of the total 1986 birth cohort (Fig. 1). In the current study, psychiatric morbidity of the participants was followed through health registers from 16 years of age until the end of the year 2018 when the participants were 33 years old.

### Data Sources

The psychiatric disorders are based on the inpatient data from the Care Register for Health Care (CRHC) until 2018, and on the data of specialized level outpatient visits available for the years 1998–2018. The CRHC has been found to be a reliable source of information in scientific research. (Miettunen et al., 2011; Gissler and Haukka, 2004). The diagnoses recorded in the CRHC are always set by physicians. The main and secondary diagnoses were taken into account in the screening of psychiatric morbidity of the study participants.

The information of parents’ mental disorders (F00–F69, F80–F99, main and secondary diagnoses) were extracted from the following nationwide registers: inpatient treatments from the Care Register for Health Care (until 2018), specialized level outpatient visits (1998–2018) and primary care (2011–2018) from the Care Register for Health Care, and information of disability pensions from the Finnish Center for Pensions (until 2016).

Participants in the cohort study and their parents were asked for signed consent to use their data for research purposes. The study obtained approval from the Ethical Review Board of the National Institute for Health and Welfare (Section 28/2009), from the Ethics Committee of Northern Ostrobothnia Hospital District (EETTMK: 108/2017) and Oulu University, Faculty of Medicine, Oulu, Finland. In addition, official permission to use individual-level register-based data for scientific research was obtained from the administration of each register.

### Measures

#### Psychiatric disorders of the study participants

The main outcomes of the current study were psychiatric disorders of the study subjects. In addition to any psychiatric disorders (ICD-10 codes: F00-F99), the following main diagnostic categories were analyzed: mental and behavioral disorders due to psychoactive substance use (F10–F19); schizophrenia, schizotypal, delusional and other non-mood psychotic disorders (F20–F29); mood (affective) disorders (F30–F39); anxiety, dissociative, stress-related, somatoform and other nonpsychotic mental disorders (F40–F49), and behavioral and emotional disorders with onset usually occurring in childhood and adolescence (F90–F98). These psychiatric diagnostic categories were chosen, because the preliminary evaluation (data not shown) showed those to be the most prevalent diagnostic categories for the purpose of the subsequent statistical analysis.

#### Social leisure time activity in adolescence

The survey for leisure time activities consisted of the following questions: How often (never, seldom, monthly, weekly, daily) do you do the following things: Meet friends, read, play, sing, recite poems, paint, write, etc., go to the movies, concerts, exhibitions, do handwork, crafts, and other similar activities, use computers, go to discos or dancing, participate in parish activities, go hiking, fishing, hunting, do scouting? Do you belong to a sports club (no; yes, but I do not attend the training sessions; yes, I attend the training sessions)? How often do you generally do the following types of physical exercise outside of school hours (never, once a month or less often, 2–3 times a month, once a week, 2–3 times a week, 4 times a week or more often)?

First, each leisure time activity was categorized to two groups based on its intensity: active leisure time (activity accomplished at least once a week) and non-active leisure time (activities accomplished less than once a week or not at all). Belonging to a sports club and participating in sports club training sessions were always defined to indicate active leisure time. Second, each activity was categorized according to the level of social activity related to it: an activity that could be done alone (etc. reading, swimming), activity where others need to be considered (etc. meeting friends, dancing, tennis), and belonging to a community (parish, scouts or sports club). After defining the intensity and level of social activity of each leisure time activity, the study participants were categorized to three hierarchical and mutually exclusive groups of social leisure activity (SLA): 1. High level of social leisure activity (high SLA), i.e. the participant belongs to a community or sports club, 2. Middle level of social leisure activity (middle SLA), i.e. the participant has leisure time activity where other people have to be considered, but he/she does not belong to a community or a sports club, and 3. Low level of social leisure activity (low SLA), i.e. the participant only has activities that can be done alone.

### Family Related Factors

Information on family structure and parents’ education was collected from a questionnaire which the parents filled in when the cohort participants were aged 15–16 years. The parents, dichotomized to those who lived together and others, were asked: Do the child’s biological parents live together? (yes; no, because they are divorced; no, because they never lived together; no, because one of the parents has died). Parental educational level (less than 9 years of comprehensive school, comprehensive school/elementary school, matriculation examination) was dichotomized to indicate at least secondary level of education completed either by the mother or the father of the study participant.

### Statistical Methods

Statistical significance of difference between groups was assessed with the Pearson Chi-square test. The association of leisure time activity (high level of social leisure activity, middle level of social leisure activity, low social leisure activity) in adolescence with diagnosed psychiatric disorders by young adulthood was analyzed with a logistic regression model. Both crude odds ratios (OR) and ORs adjusted for gender, biological parents living together, parental education level and parental psychiatric disorders are reported in the results.

Figure 1 shows the selection of the data for the current study. Initially, the 1986 Northern Finland Birth Cohort consisted of 9423 subjects. For the follow-up survey completed at the age of 15–16 years, 9215 (97.8% of initial population) cohort members had a known mailing address. Of them, 7344 (79.7% of eligible sample) returned the postal survey completed at the age of 15–16 years. The attrition analysis made in the follow-up survey at age 15–16 years showed that participation in the survey was less common among males (participants vs. attrition sample) (64 vs. 71%, *p* < 0.001), those living in urban areas (66 vs. 71%, *p* < 0.001) and those having a parent with a psychiatric disorder (58 vs. 69%, *p* < 0.001) (Miettunen et al., 2013) In the follow-up survey, questions related to leisure time activities were answered by 7283 (99.2% of survey participants) cohort members, of whom 7051 (96.0% survey participants) gave permission to use their data for research purposes. Of them, 213 were excluded due to mental disorder diagnoses set before 16 years of age. Furthermore, 872 participants had missing data on family-related factors (parents living together, etc.), and the final size of the study population in the statistical analyses was 5966 participants. The number of cases dropped from the adjusted model (in comparison to data available for SLA categorization) was 413 among males and 459 among females (12.5 v. 13.0%, *p* = 0.583), 345 among individuals with parental psychiatric disorders, and 527 among individuals without parental psychiatric disorders (13.7 v. 12.2%, *p* = 0.086).

Statistical analysis was performed using SPSS Statistics 25.

## Results

Of all study participants, 2188 (32%) belonged to high level of social leisure activity (high SLA) group, 4502 (65.8%) to middle level of social leisure activity (middle SLA) group, and 218 (3.2%) to low level of social leisure activity (low SLA) group.

As Table 1 shows, of all study participants, 48.2% were males and 51.8% were females. As for parental characteristics, 78.3% of the participants had biological parents living together, and 38.0% of the parents had completed at least secondary level education. A total of 37.0% of the participants had at least one parent with a history of doctor-diagnosed psychiatric disorder.

**Table 1:** Psychiatric disorders by young adulthood and background characteristics of the study participants in relation to leisure time activity in adolescence, the 1986 Northern Finland Birth Cohort Study

	Total		Level of social leisure activity						
			Low-SLA^a^ (*n* = 218)		Middle-SLA^a^ (*n* = 4502)		High-SLA^a^ (*n* = 2118)		
	*n*	%	*n*	%	*n*	%	*n*	%	*p*-value
Gender									
Male	3298	48.2	104	47.7	2070	46.0	1124	53.1	*<0.001*
Female	3540	51.8	114	52.3	2432	54.0	994	46.9	
FAMILY RELATED FACTORS Biological parents living together^b^									
No	1299	21.7	45	23.3	905	23.3	349	18.3	*<0.001*
Yes	4687	78.3	148	76.7	2985	76.7	1554	81.7	
Parental education^b^									
Comprehensive school or less	3781	62.0	120	61.2	2641	66.6	1020	52.7	*<0.001*
Secondary school graduate	2316	38.0	76	38.8	1326	33.4	914	47.3	
Parental psychiatric disorder^c^									
No	4312	63.1	134	61.5	2787	61.9	1391	65.7	*0.011*
Yes	2526	36.9	84	38.5	1715	38.1	727	34.3	
PSYCHIATRIC DISORDERS									
F00-F99: Any psychiatric disorder	1003	14.7	49	22.5	704	15.6	250	11.8	*<0.001*
F10-F19: Substance use disorders	206	3.0	8	3.7	154	3.4	44	2.1	*0.010*
F20-F29: Psychotic disorders	97	1.4	4	1.8	67	1.5	26	1.2	0.613
F30-F39: Affective disorders	522	7.6	25	11.5	378	8.4	119	5.6	*<0.001*
F40-F49: Anxiety	539	7.9	33	15.1	379	8.4	127	6.0	*<0.001*
F90-F98: Behavioural disorders	80	1.2	8	3.7	51	1.1	21	1.0	*0.002*

Participant can have diagnosis from several diagnose group. Levels of social leisure activity are mutually exclusive

^a^*SLA* social leisure activity

^b^When cohort members were 15–16 years old

^c^Until the end of 2018

The results of comparisons between the three SLA groups showed higher prevalence of males in the high SLA group (53.1%) compared to the low SLA (47.7%) and middle SLA (46.0%) groups (*p* < 0.001). The proportion of biological parents not living together was significantly lower in the high SLA group (18.3%) compared to 23.3% in both the low- and middle SLA groups (*p* < 0.001). The proportion of parents who had completed at least secondary level education was lowest in the low SLA (38.8%) and middle SLA (33.4%) groups compared to the high SLA (47.3%) group (*p* < 0.001). The parental psychiatric disorders were less common in the high SLA group (34.3%) compared to the low SLA (38.5%) and middle SLA (38.1%) groups (*p* < 0.001).

The incidence of psychiatric disorders diagnosed for the first time when the study participants were 16–33 of age was 14.7%. The most common diagnostic groups were anxiety (7.6%), affective (7.9%) and substance use (3.0%) disorders. The level of SLA was statistically significantly associated with the incidence of any psychiatric disorder (*p* < 0.001), substance disorders (*p* = 0.010), affective disorders (*p* < 0.001), anxiety disorders (*p* < 0.001) and behavioral disorders (*p* = 0.002). Generally, the incidence of these psychiatric disorders was highest in the low SLA group and lowest in the high SLA group.

Table 2 shows the results of logistic regression analyses examining the association of social leisure time activity in adolescence with likelihood of physician-diagnosed psychiatric disorder by young adulthood. Low SLA was associated with increased likelihood of any psychiatric disorder (OR 1.61, 95% CI 1.13–2.30), anxiety disorders (OR 2.07, 95% CI 1.37–3.15) and behavioral disorders (OR 4.12, 95% CI 1.90–8.96). Furthermore, high SLA was associated with decreased likelihood of any psychiatric disorders (OR 0.78, 95% CI 0.66–0.93), substance use disorders (OR 0.53, 95% CI 0.36–0.78), affective disorders (OR 0.72, 95% CI 0.57–0.91) as well and anxiety disorders (OR 0.79, 95% CI 0.63–0.99). Social leisure time activity was not associated with psychotic disorders of the study participants.

**Table 2:** Association of leisure time activity in adolescence with likelihood of psychiatric disorder diagnosed by young adulthood, the 1986 northern finland birth cohort study

	Likelihood for psychiatric disorder (outcome)^a^				Statistically significant covariates in adjusted model (OR and 95% CI)
	Low-SLA^b^		High-SLA^b^		
Outcome variable	OR	95% CI	OR	95% CI	
F00–F99: Any psychiatric disorder					
Crude model	1.56	*1.13*–*2.17*	0.72	*0.62*–*0.84*	Gender (1.40, 1.20–1.62), Biological parents living together (1.53, 1.30–1.80), Parental psychiatric disorder (1.74, 1.49–2.01)
Adjusted model	1.61	*1.13*–*2.30*	0.78	*0.66*–*0.93*	
F10–F19: Substance use disorders					
Crude model	1.08	0.52–2.22	0.60	*0.43*–*0.84*	Gender (0.53, 0.37–0.70), Biological parents living together (2.25, 1.64–3.08), Parental psychiatric disorder (1.91, 1.40–2.61)
Adjusted model	1.21	0.58–2.52	0.53	*0.36*–*0.78*	
F20–F29: Psychotic disorders					
Crude model	1.24	0.45–3.42	0.82	0.52–1.30	Gender (0.60, 0.38–0.92), Parental psychiatric disorder (2.14, 1.38–3.31)
Adjusted model	1.34	0.48–3.74	0.79	0.49–1.29	
F30–F39: Affective disorders					
Crude model	1.41	0.92–2.17	0.65	*0.52*–*0.80*	Gender (1.91, 1.55–2.34), Biological parents living together (1.55, 1.25–1.93), Parental psychiatric disorder (1.85, 1.52–2.26)
Adjusted model	1.45	0.91–2.31	0.72	*0.57*–*0.91*	
F40–F49: Anxiety disorders					
Crude model	1.94	*1.32*–*2.85*	0.69	*0.56*–*0.85*	Gender (1.63, 1.33–1.99), Biological parents living together (1.56, 1.26–1.93), Parental psychiatric disorder (1.77, 1.45–2.15)
Adjusted model	2.07	*1.37*–*3.15*	0.79	*0.63*–*0.99*	
F90–F98: Behavioural disorders					
Crude model	3.32	*1.56*–*7.10*	0.87	0.52–1.46	Biological parents living together (2.19, 1.33–3.61), Parental psychiatric disorder (1.73, 1.06–2.83)
Adjusted model	4.12	*1.90*–*8.96*	1.12	0.65–1.93	

OR’s calculated with logistic regression with several outcomes. Crude model and model adjusted with gender, family structure and parental education when cohort members were 15–16, and parental psychiatric disorders until the end of 2018, has been built to examine associations between hierarchical hobby and psychiatric disorders. Participant can have a diagnosis from several diagnosis groups. Levels of social leisure activity are mutually exclusive

^a^Reference category = Middle social leisure activity

^b^*SLA* social leisure activity

## Discussion

Leisure time activities are known to have a positive effect on mental health. However, previous studies of this association have focused mainly on mental health symptoms but not on psychiatric disorders. Thus, in this respect, the research-based evidence of the impact of leisure time activities on psychiatric morbidity is still limited. This type of knowledge is needed to focus and to develop preventive actions for mental disorders. In this study, it was possible to analyze at the population level whether socially different leisure time activities during adolescence associate with first-onset mental disorders diagnosed between ages 16–33 years.

The main finding of the study was that socially active leisure time (high SLA) in adolescence was associated with a lower incidence of psychiatric disorders. Analogously, socially inactive leisure time (low SLA) was related to increased occurrence of psychiatric disorders. These findings remained significant even after controlling for commonly known family-related risk factors for psychiatric morbidity of young individuals, such as parents’ mental health disorders (Merikukka et al., 2018), parental educational level (Paananen et al., 2013), and living with a single parent (Amato, 2000). Thus, the findings of the current study can be interpreted to underline the importance of community in leisure activities as a protective factor against mental disorders. This is in line with previous studies reporting that community sport participation with improved physical health also has a positive effect on mental health among young people, seen as increasing positive psychological well-being and improving social health (e.g. Rochelle et al., 2013).

In this study, of the specific psychiatric disorders, socially active leisure time (high SLA) was found to decrease the likelihood for anxiety and affective disorders. One plausible explanation for this finding could be the role of environmental factors, which may either protect against or promote the development of these disorders (e.g. Beesdo et al., 2009). Epidemiological survey findings have also shown that anxiety disorders are the most prevalent mental disorders worldwide and are associated with significant comorbidity and morbidity (Stein et al., 2017). The development of depressive symptoms or anxious reactions to a diagnosed psychiatric disorder is usually a longer process. Commonly, it includes accumulation of various adverse experiences in everyday life of the adolescent. On the other hand, this time frame enables an individual to get support from the community, and consequently, to gather positive, rehabilitating experiences which may have a preventive impact on the development of potential mental disorders. The social interactions in the community can also provide alternative perspectives on stressors and challenges and give collaborative solutions for the difficulties an individual may have in his/her everyday life. Previous studies have acknowledged that on the individual level, interactions with close people within the community increase a sense of togetherness and lead to self-confidence and social skills (e.g. Ungar, 2012), which strengthens individuals’ mental health.

An important finding of this study was that high SLA was related to decreased likelihood of substance use disorders. In earlier studies, community-based activity, such as sports clubs, are shown to reduce the likelihood for substance abuse (Kristjansson et al. 2010). This can be seen as a result of the positive example of the community and the meaningful activity the community offers as an alternative to substance abuse. Also, the social pressure of an organized activity group and its rules of prohibiting substance use may affect individuals’ behavior. Overall, the high SLA findings of the current study give further support to the impact of leisure time activities on the positive mental health of young persons (e.g. Agans et al., 2014).

Socially inactive leisure time (low SLA) increased the incidence of anxiety and behavioral psychiatric disorders. One explanation for an excess of anxiety disorders might relate to the individuals themselves; for example, they may have a sensitive and introvert personality and therefore seek out leisure time activities that can be done alone (Whisman et al., 2000). Because of hobbies that are not social, adolescents may also have missed out on the potential protective effect of communities. Also, on community level, it is possible that some peer groups do not accept anxious or introverted persons as a group member. Accumulated experiences of being alone and left outside peer groups can be a source of anxiety and other mental health problems (Cantor-Graae and Selten, 2005). In this study, behavioral disorders were also associated with socially inactive leisure time. This may be due to decreased social capability, which is one typical characteristic of the disorder (Ogundele, 2018). Thus, the symptoms of these behavioral disorders may have been affecting the ability to be part of or to be approved by a peer group. This same phenomenon might be a plausible explanation for inactive social leisure time with other psychiatric disorders as well.

The current study showed that first-onset psychotic disorders did not associate with either high or low social leisure time activity, although the occurrence of psychotic disorders was in line with general population rates (Moilanen et al., 2010). The explanation may be that psychiatric morbidity cannot be explained only by social or environmental factors, but also by genetic transmission (e.g. Kendler et al., 2003). The role of genetic factors is emphasized especially among the most severe disorders, such as psychosis (Holmans, 2020). However, in terms of preventing these disorders and according to the stress-vulnerability model (Myllyaho et al., 2021), meaningful social leisure time activities could be beneficial in reducing stress and providing tools to cope with them.

The present study has several strengths. Firstly, the cohort data is extensive and covers the entire birth cohort in Northern Finland. The follow-up of the subjects is based on register data that is monitored. Secondly, the Finnish Care Register has been found to be a reliable source of information in studies (Gissler and Haukka, 2004). In this study, it was possible to access the psychiatric diagnosis-related information far into adulthood. The registers utilized here provided the possibility to follow the accumulation of first onset of mental disorders occurring from 16 up to 33 years of age among the cohort members. The third strength of this study is that mental disorder diagnoses were extracted from the healthcare registers including all inpatient hospitalizations and specialized level outpatient visits of the study participants nationwide. The diagnoses in health care registers are always based on the ICD classification of diseases, which uses operational diagnostic criteria. Diagnosing a mental disorder always requires a thorough clinical examination (assessment of psychiatric status) of a patient, including differential diagnostics, not only between the psychiatric disorders, but also in relation to somatic illnesses. The clinical evaluation includes assessment of symptom criteria, not only symptoms informed by the patient, but also symptoms objectively observed by health care professionals. The severity (impact on distress and performance) and duration of the symptoms are also crucial criteria for mental disorder diagnosis. Research utilizing data on self-reported symptoms is often prone to subjective bias and, thus, caseness determination, i.e. a condition or fact the diagnostic criteria for a psychiatric disorder are satisfied, is unreliable.

There are some limitations attached to this study. The first limitation is that some mental disorder diagnoses were made by treating physician, not by a mental health specialist (psychiatrist), causing bias in the assessment of specific diagnosis of mental disorder. They have differences in their clinical practices because of differences in training and working experience. The second limitation in this study is that there is no information on whether structured methods have been used to set the diagnoses and also, it is not possible to control diagnostic practices between physicians. The third limitation of this study is the lack of information about undiagnosed mental health issues of the study participants. Some of the study participants’ mental health problems may have remained unrecognized and untreated in youth and the results can thus be biased due to undiagnosed mental health issues. The fourth limitation is that the small number of cases in the subgroup analyses may have caused lack of power in statistical analyses (Type II error). In addition, due to the many statistical comparisons performed in this study the possibility of chance findings (Type I error) cannot be excluded.

## Conclusion

Research-based knowledge on whether the level of adolescent social leisure time activity is associated with specific physician-diagnosed mental disorders already by young adulthood has been limited. In this study, the main findings were that socially active leisure time in adolescence was associated with a lower incidence of psychiatric disorders in general, and, specifically, with affective, anxiety, and substance use disorders by young adulthood. Analogously, low social leisure time activity was related to increased incidence of any psychiatric disorder in general, and to anxiety and behavioral disorders in particular. Overall, our findings strongly indicate that socially active leisure time during adolescence is a protective factor against development of later psychiatric disorders. Social support of the community in the early phase is one way to prevent development of mental health symptoms into manifest psychiatric disorders. In terms of prevention of mental disorders, these study findings encourage families, schools and other communities to continue to enhance and develop community-based social leisure time activities for children and adolescents.
